# MRI safety—developing the right culture

**DOI:** 10.1007/s00247-025-06179-5

**Published:** 2025-02-14

**Authors:** Dana Alkhulaifat, Lorenna Vidal, Ethan Larsen, Suraj D. Serai, Mario Sinti-Ycochea, Patricia Mecca, Lauren Orfe, Susan Sotardi

**Affiliations:** 1 Department of Radiology, Children’s Hospital of Philadelphia, 3401 Civic Center Blvd, Philadelphia, PA 19104 USA; 2 University of Pennsylvania, Philadelphia, PA USA

**Keywords:** Magnetic resonance imaging, MRI safety, Just Culture approach

## Abstract

In recent years, magnetic resonance imaging (MRI) among pediatric populations has risen, requiring strict adherence to MRI safety protocols for patient care. Children often face more complex safety risks compared to adult populations due to several challenges, such as increased need for sedation, and the limited data and testing on implant safety in pediatric patients. Our aim is to examine the key features of MRI safety and how safety culture impacts important aspects of MRI processes, including patient and staff screening, physical barriers, zoning regulations, emergency response protocols, and adverse event management. This article also explores the cornerstone of MRI safety, the Just Culture approach, which emphasizes transparency, accountability, and improvement of processes over punishment. Key efforts towards building and maintaining safety culture focus on raising awareness, outlining escalation procedures, and instilling a safety-first mindset. Given MRI safety’s critical importance in pediatric patient care and staff well-being, the development of a culture that supports these goals is an imperative for every imaging center.

**Graphical Abstract:**

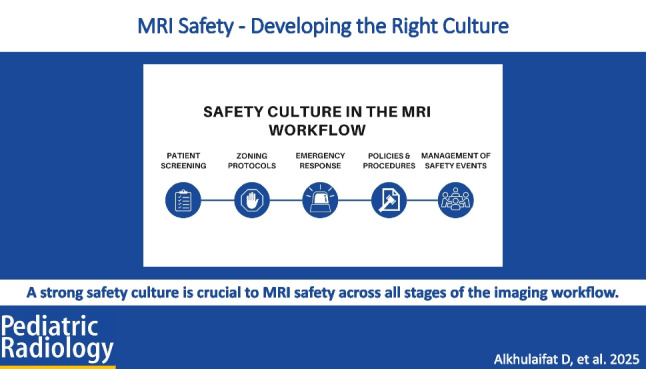

**Supplementary Information:**

The online version contains supplementary material available at 10.1007/s00247-025-06179-5.

## Introduction

With the rise in magnetic resonance imaging (MRI) in the pediatric population over the past two decades, safeguarding children undergoing MRI procedures has emerged as a top priority [1]. While MRI may provide benefit for children due to the elimination of ionizing radiation, studies indicate that MRI safety risks occur at a higher rate in children compared to adults. This is due to the fact that MRI introduces its own unique set of risks, including torque and translational forces leading to lethal projectiles, which tragically led to the death of Michael Colombini in 2001, and thermal injuries, a common MRI-related injury [2–4]. Pediatric populations represent the largest demographic requiring sedation during MRI procedures, thereby increasing their risk profile for adverse events [5]. Children are also more susceptible to developing anxiety during an MRI scan and may experience difficulties in following verbal commands or expressing their concerns. Thus, a focused MRI safety plan must be in place to support children, parents, or other healthcare providers present in the MRI environment during the imaging procedure [6, 7]. However, safety protocols, policies, and directives are often unsuccessful when a culture of MRI safety is not fostered among the stakeholders. This culture calls for adherence to stringent safety protocols, while establishing a mindset that prioritizes vigilance, collaboration, and continuous improvement in all aspects of MRI operations [8].

Establishing a successful MRI safety initiative hinges on an organized structure with well-defined roles and responsibilities [8, 9]. The Inter-Society Working Group on MR Safety published recommendations for an appropriate organizational structure for MRI safety [10]. The MRI Medical Director (MRMD) is typically a radiologist who oversees the MRI safety operations. The MRMD works alongside the MRI Safety Officer (MRSO) and MRI Safety Expert (MRSE) to structure safety protocols, supervise their implementation, and ensure adherence to regulations and guidelines [11]. The MRSO, typically a senior MRI technologist, implements safety procedures, educates staff, and reports safety issues. The MRSE, an MRI physicist, provides expert guidance on equipment safety and protocol modifications [12, 13]. This collaborative effort forms the core of MRI safety, representing the primary point of contact for all MRI safety issues [10]. The MRI safety initiative is further strengthened by the participation of a wider MRI safety committee, comprising key stakeholders (the MRMD, MRSO, and MRSE) in addition to representatives from various departments. Regular meetings are essential for reviewing and updating policies, discussing workflow optimization, and reporting safety events [7].

Ultimately, the goal of establishing an MRI safety culture is to ensure that safe practices are upheld by everyone by facilitating open and transparent communication channels between all stakeholders [7]. The establishment of MRI safety culture relies on adherence to regulations such as the American College of Radiology’s (ACR) Manual on MRI Safety, which sets the standard for safe MRI environments [14], and maintaining accreditation mandated by the Medicare Improvements for Patients and Providers Act to ensure facilities uphold strict safety and quality standards [15]. By creating a culture that prioritizes MRI safety throughout a health organization, providers can effectively mitigate safety risks associated with MRI use in children.

In this article, we explore important aspects of MRI safety and the ways in which safety culture influences each stage of the MRI workflow. From the initial screening of patients, to implementation of physical barriers, scanning procedures, emergency response protocols, policy development, and adverse event management, the culture within an MRI facility plays a pivotal role in determining safety outcomes [7].

## Understanding the culture of safety

The traditional perspective on organizational safety focuses on attributing errors to the actions of individuals [16]. In this outdated framework, the primary objective is to identify and address problematic individuals with remediation or punishment, which consequently leads to a decrease in error reporting. In contrast, research now shows that MRI safety is more effective when organizations shift their perspective towards mitigating external processes which hinder safe behaviors [17]. Errors are seen as outcomes of systemic failures within the MRI environment. Consequently, error analysis is more holistic and looks at the circumstances that led to human error [16].

A crucial component in ensuring a culture of MRI safe practices lies in the commitment of institutional and departmental leaders, in addition to those who are permanently or transiently involved in the MRI environment, such as patient care teams [18]. Radiology department leaders play a pivotal role in achieving MRI safety by consistently striving to identify key aspects for safety improvement. The establishment of MRI safety leadership provides accountability and support which is usually achieved with MRI safety committee, ensuring regular meetings to identify safety risks and near misses, and devising strategies to prevent future incidents. These efforts must be collaborative and supported at an institutional level to increase awareness of MRI safety practices across various hospital departments, where differing levels of knowledge about MRI safety practices may exist. Finally, at an individual level, safety must be understood as paramount for all MRI personnel, with integration into routine operations. Achieving the goals demands regular educational sessions on MRI safety practices and reinforcing learning through interactive simulations of common safety risk scenarios, thereby enabling effective preparation for real-life situations [19].

## Patient screening

Screening is a fundamental step in ensuring a safe MRI workflow for both patients and personnel [6]. The known risks associated with the presence of ferromagnetic objects in the MRI environment, which endanger both the patient and surrounding personnel, call for an expeditious screening protocol that aims to identify metallic objects, whether external or implanted, before patient and personnel enter the scanner room [20]. Screening processes and forms should be consistent for patients, non-MRI personnel, and MRI personnel. Templates for MRI screening forms can be accessed through the ACR safety website [7].

At our institution, screening commences once the imaging order is placed. During order entry, the ordering clinicians can identify if the patient has an implant. Then, prior to scheduling the MRI, the outpatient schedulers perform a pre-screen utilizing a standard list of potentially hazardous devices that should be investigated and cleared (provided in Supplementary File 1). The patient is scheduled for an exam if no device is found. On the day of the imaging, the patient undergoes additional screening upon arrival at the radiology department. MRI personnel are advised to question the patient both with and without the presence of a parent or guardian, as pediatric patients are not accurate medical historians. Furthermore, safety precautions, such as changing to an MRI-safe gown and discouraging personal belongings (e.g., stuffed animals), can prevent the inadvertent introduction of metallic objects or other hazards into the MRI environment. The MRI technologist, who is the expert in the room, is responsible for performing the final check, which includes confirming patient identification and completing the MRI safety screening for the patient, support equipment, and personnel immediately prior to entering the MRI room [6, 14]. When active or passive implants and device are detected, the MRI technologist will perform assessments of its safety profile before proceeding with the MRI. In an outpatient setting, the assessment is conducted days before the scheduled MRI, initiated by information provided during the scheduling process. However, in emergency and inpatient settings, the assessment takes place immediately after the request or order is placed. The process involves chart review, research on device MRI compatibility, and direct communication with vendor liaisons to determine whether the implant, foreign body, or device is MRI-safe or conditional. If the MRI technologist cannot clear the device, the MRSO steps in and may consult with the MRMD and MRSE for a more detailed risk assessment and clearance [6]. In situations where the MRI conditions for implants, foreign bodies, or devices cannot be met, are unknown, or are deemed unsafe, the MRMD is responsible for evaluating the risks and benefits. They must decide whether to cancel the MRI study or proceed with precautions to mitigate the risk of injury [21].

Each step in the screening process requires attention to detail and open communication channels, from initial instructions provided by schedulers to verification by technologists and nurses upon the patient’s arrival. As the patient transitions through various stages of MRI preparation, thorough handoffs between personnel are imperative to ensure that all concerns are addressed and that no information is lost. Successful communication strategies between different professional groups include closed-loop communication, which has demonstrated effectiveness in high-stress environments, such as in pediatric emergencies and resuscitations [22]. Deferring to expertise is an essential practice in high-reliability organizations and reflects the recognition and deference to the most knowledgeable and skilled person in the room, which in the MRI environment is the MRI technologist [23]. Another vital component of successful screening is creating an environment where everyone feels empowered to speak up when they notice potential risks, thereby fostering a safer environment for patients and staff alike [18]. Therefore, to enable individuals to state their concerns, there must be a culture where respect and equity are promoted among all workers. This culture needs to be supported by the institution, in order to challenge the historically hierarchical culture of a healthcare organization.

## Zoning regulations and physical barriers

Zoning regulations further contribute to MRI safety, providing physical environmental controls to ensure that areas leading into the MRI suite are restricted to MRI personnel and certified non–MRI personnel who underwent adequate training. According to the ACR, non-MRI personnel are individuals who have not completed formal MRI safety education required by the facility’s MRMD within the past 12 months. In contrast, MRI personnel are those who have undergone specialized safety training. These regulations prevent unauthorized access and minimize the risk of accidents [8, 14]. The ACR safety guidelines classify MRI personnel into two categories: Level 1 and Level 2 personnel, who receive different levels of training based on their roles in the MRI environment. Level 1 personnel complete the facility’s MRI safety training requirements, as set by the MRMD. In comparison, Level 2 personnel receive more extensive training due to the demands of their roles in the MRI environment. Teams that frequently work in this setting, such as those in anesthesia and sedation, often have Level 2 access. Entry into the MRI environment necessitates completing designated training modules, which grant them badge access to the MRI area. In addition, regular MRI safety training ensures that personnel stay updated on important policy and procedure changes while maintaining their certifications [14].

**Fig. 1:**
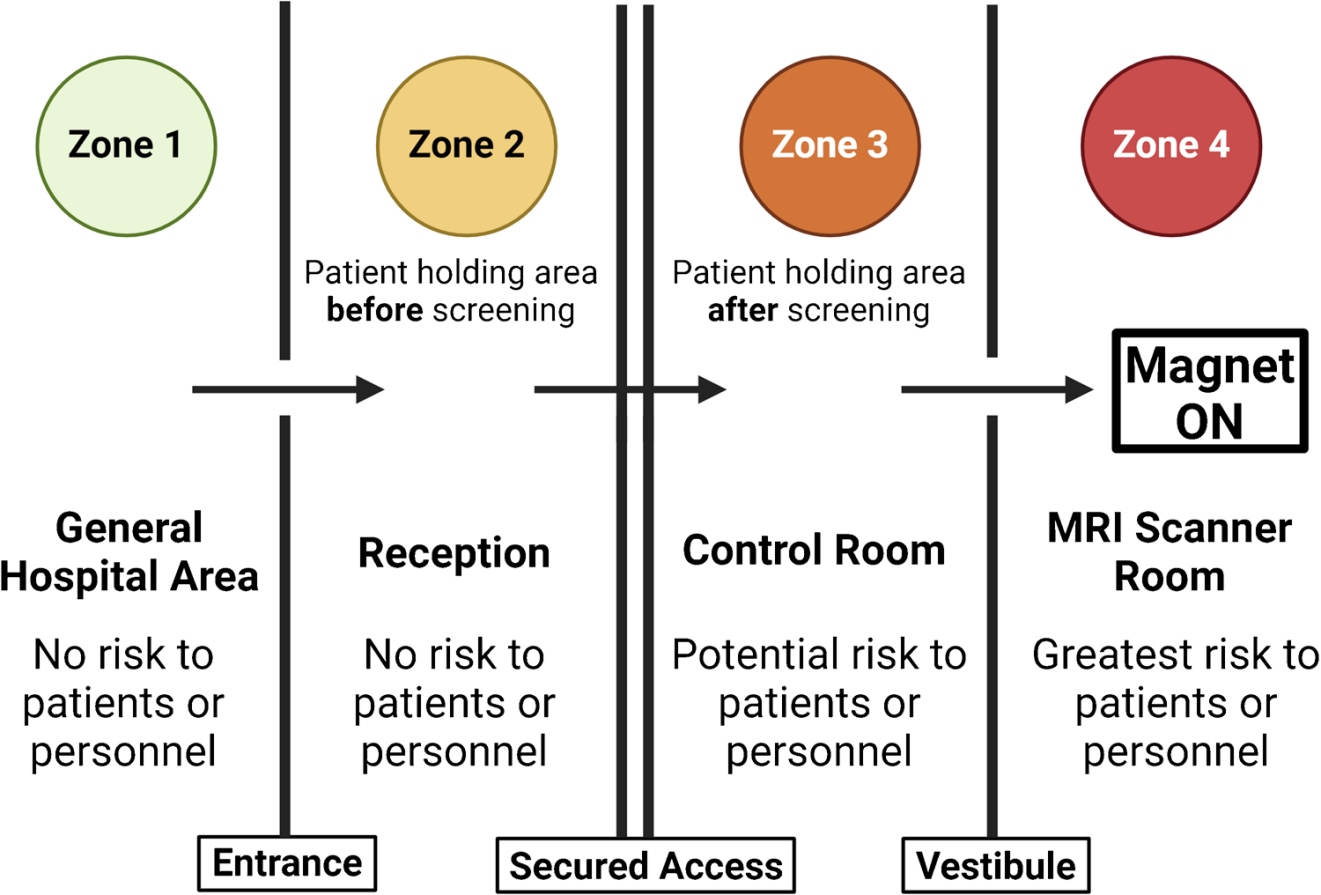
The four MRI safety zones outlined by the American College of Radiology, organized by their proximity to the MRI magnet. Each zone is assigned a different *color* to signify the varying security requirements and associated risk levels

The ACR delineates four safety zones within MRI facilities, each with variable levels of access restrictions according to their associated risk, as shown in Fig. 1 [5]. Zone I encompasses areas outside the MRI environment that are open to the general public. Zone II forms the patient care area between Zone I and the more restricted zones (III and IV), and is where the patient is first introduced to the MRI environment. Within this area, patient preparation is performed, which includes screening, obtaining medical history, and gowning. Zone III contains the imaging control unit. Access to Zone III is tightly controlled by higher level MRI personnel, and physical restrictions prevent general public access such as key locks or passkey systems. Non-MRI personnel, even those certified, must always be accompanied by the MRI technologist within Zone III. Due to Zone III’s proximity to Zone IV, which may occasionally fall outside the technologist’s line of sight, it should be treated with the same level of caution as Zone IV. Zone IV comprises the scanner room itself and represents a critical area where severe injury or potential death can occur from the introduction of ferromagnetic objects, and entrance to this area must display prominent signage indicating that the MRI magnet is always on. Any movement from Zone III into Zone IV requires verbal authorization by the MRI technologist and involves passing through a physical barrier to avoid unintended passage of unauthorized personnel, which serves as the last checkpoint preventing the introduction of ferromagnetic material. To maintain Zone IV security, high-level MRI personnel, such as MRI technologists, maintain constant observation of access points through either direct observation or video monitoring [8, 14].

While regulations are provided by the ACR governing how movements should be controlled through the space, actual implementation of the recommendations is often inconsistent, even within the same institution. Utilizing fundamental interface design heuristics common in healthcare recommendations can be made for ACR-compliant space design [24, 25]. For example, the zone signs should be of sufficient size to be read and noticed by all personnel, with lay language to ensure non-MRI trained personnel can understand the safety concerns of the space. Colors and symbols can also be utilized but consistency is key. In our institution, we are utilizing green for Zone I, yellow for Zone II, orange for Zone III, and red for Zone IV. In addition to aligning all signs to these colors, we are altering the environments to use those same colors in flooring, paint, and wall graphics to help reinforce that the environment is different and distinct from a standard hospital space, and the color progression relates to the increasing security needs of the space.

The implementation of zones offers a fundamental framework for regulating activities in the MRI environment. However, the process of regulating these zones underscores the importance of respect for established safety protocols, with MRI technologists serving as the clear authorities in enforcing zone regulations and maintaining the MRI safety environment. Therefore, MRI technologists need empowerment by the leadership to enforce regulations and safety in the MRI environment.

## Emergency response

Patient-related emergencies demand multifaceted preparedness strategies. MRI Zone IV limits resuscitation interventions; therefore, in the event of a cardiac arrest, the main goal is to remove the patient from Zone IV immediately for cardiopulmonary resuscitation [7]. However, long before an emergency occurs, any non-MRI personnel expected to participate in resuscitation teams must be screened and trained in the MRI environment. Consistency in training, through regular simulations, has been shown to improve emergency preparedness [19]. Additionally, any essential equipment should be verified as MRI safe or MRI conditional, and strategically placed for easy access by the clinical care team performing the resuscitation. While devices are clearly labeled for use in the MRI environment, we encourage a culture of practicing with a questioning attitude for our technologist staff to ensure that any potential piece of equipment coming into the MRI area is evaluated in its entirety. By anticipating the needs of the code team, MRI safety personnel facilitate the protection of the patient and all involved personnel [13].

In situations demanding immediate cessation of MRI operations and magnet quench, the preparation of MRI personnel is essential. An MRI emergency action plan outlining comprehensive steps should be established, which includes initiation of magnet quench, safe evacuation of patients and personnel, and mitigation of risks associated with magnet quench, such as asphyxiation and cryogenic burns due to leak of low-temperature gas into the MRI room [8]. In these scenarios, effective preparation and communication between personnel decrease the likelihood of adverse events occurring during emergencies.

## Policies and procedures

Establishing a culture that prioritizes safety improvement allows MRI facilities to adapt dynamically to evolving needs and continuously enhance their safety processes [14]. Once policies and procedures for MRI safety are established, regular review and updates must be conducted. Led by the MRI Safety Council and supported by the larger body of stakeholders in the MRI Safety Committee, the review process should identify potential gaps in day-to-day operations. By continuously remaining vigilant for potential areas of improvement and respecting feedback from stakeholders, MRI facilities can incorporate important perspectives into their safety protocols. Policies should incorporate the ACR Committee on MRI Safety’s recommendations on reporting of adverse events, near misses, incidents, and equipment malfunctions, in accordance with United States Food and Drug Administration guidelines. Therefore, the process of updating policies and procedures for MRI safety relies upon a culture of transparency and respect [14].

## Management of safety events

Root cause analysis (RCA) is an essential step in the management of safety events in healthcare settings. According to the Joint Commission, a patient safety event refers to an incident which had the potential to or did cause harm to a patient. When a safety event does result in harm, it is classified as an adverse event. Sentinel events, a subcategory of adverse events, are situations that result in severe outcomes, such as death, severe injury, or permanent harm, regardless of the patient’s underlying condition. In contrast, a “near miss” is an event that had the potential to cause harm but was averted before any harm occurred. In the event of a major safety incident, RCA meetings can identify the underlying systemic causes that led to the event, enabling the development of effective strategies to mitigate future risks [26–28]. The Joint Commission mandates that a comprehensive systematic analysis be conducted within 45 days for major or sentinel adverse events in the clinical environment [29]. In RCA, a multidisciplinary team is assembled to investigate the incident, with an emphasis on identification of system-wide factors rather than individuals. The aim is to isolate all contributing factors to the error and create risk mitigate plans. These “root causes” may include problems in communication, environmental issues, inadequate or ineffective training, and impractical policies [16].

A robust safety culture enables and encourages individuals to disclose errors. Alternatively, ineffective safety cultures create barriers for reporting and incentivize error concealment. Punitive measures for incident reporting, hesitancy to challenge authority, and concerns about negative repercussions on the work environment have all been cited as reasons for concealing errors [30]. Additionally, fear of litigation and past experiences of unfair punishment also cause individuals to avoid reporting errors.

The Just Culture approach prioritizes safety improvement over punitive measures [31]. The process operates under a no-blame methodology, which shifts the emphasis from blaming individuals to emphasizing accountability. Just Culture assesses safety events based on intent, rather than outcome, without absolving individuals of responsibility for the error. It provides a structured approach aimed at understanding circumstances that led to the error. Adopting this approach provides the foundation for the improvement of safety culture in radiology departments. Demonstrable safety improvement has been observed at institutions after Just Culture algorithms were incorporated into practice [32]. Prior studies emphasize the importance of creating an environment where individuals feel safe and empowered to disclose errors. Furthermore, this environment has been shown as a robust predictor of improved outcomes in healthcare settings [33].

In the Just Culture framework, the manner in which safety issues are presented and discussed, throughout the organization, directly influences the outcome of the process. Preserving privacy and confidentiality of involved individuals mitigates the defensive posture that encourages error concealment. Furthermore, discussions about errors should be approached with empathy and understanding in order to support the involved individuals. In doing so, team members learn that they will be treated fairly in the event of an error, thereby lowering the barriers for discussion [32].

Despite the demonstrable benefit, the implementation of Just Culture in radiology may encounter challenges. Successful implementation relies on commitment from organizational leaders. Trust in the consistent execution of this new approach may require time, particularly if leaders and personnel have historical reasons for erosion in mutual confidence [32]. Promoting open discussion and changing the way radiology leadership responds to errors are essential to this cultural shift, but require sustained efforts [18]. Radiology and institutional leadership must therefore recognize that adopting and supporting these practices is a necessary investment in MRI practice [29].

## Conclusion

In conclusion, MRI safety is essential in maintaining the well-being of patients and staff. The culture surrounding the processes and procedures of MRI safety has the potential to either support or derail imaging operations. Evidence suggests that the Just Culture approach, which emphasizes transparency, accountability, and process improvement, fosters a supportive safety culture, mitigates risk, and improves outcomes. In essence, MRI safety culture is not merely a set of rules and regulations, but a mindset focused on shared commitment to prioritizing safety in pursuit of better healthcare outcomes.

## Supplementary Information

Below is the link to the electronic supplementary material.

Supplementary file1 (PDF 2781 KB)

## Data Availability

No datasets were generated or analyzed during the current study.
