# Supplementary material for: MRI safety—developing the right culture
Source: Pediatr Radiol. 2025 Feb 14;55(5):895–901. doi: 10.1007/s00247-025-06179-5 (PMC12065676; doi:10.1007/s00247-025-06179-5)
Supplement: Supplementary file 1 — Supplementary file1 (PDF 2781 KB) [file 247_2025_6179_MOESM1_ESM.pdf]

OUTPATIENT MAGNETIC RESONANCE IMAGING (MRI) PROCEDURE SCREENING

This form is to be completed by a patient/parent/patient representative.

Prior to the patient having an MRI, he/she will need to be checked for metallic items on or within the body. Even something as simple as lotion with glitter can cause the patient harm. We care about the safety of the patient and need you to take a few minutes of your time to complete this form.

1. Has the patient experienced any problem related to a previous MRI? ☐ Yes ☐ No ☐ Unknown
   If yes, please describe: ______________________________ ☐ First MRI
2. Has the patient had an injury to the eye involving a metallic object or fragment (e.g., metallic slivers, shavings, foreign body, etc.)? ☐ Yes ☐ No ☐ Unknown
   If yes, please describe: ______________________________
3. Has the patient ever been injured by a metallic object or foreign body (e.g., BB, bullet, shrapnel, etc.)? ☐ Yes ☐ No ☐ Unknown
   If yes, please describe: ______________________________
4. Does the patient have anemia or any disease(s) that affects his/her blood, a history of renal (kidney) disease, or seizures? ☐ Yes ☐ No ☐ Unknown
   If yes, please describe: ______________________________

For menstruating female patients:

5. Date of last menstrual period:____/____/____ ☐ Yes ☐ No ☐ Unknown

***Please indicate if the patient has any of the following:**

Aneurysm clip(s)	☐ Yes	☐ No	☐ Unknown
Cardiac pacemaker	☐ Yes	☐ No	☐ Unknown
Implanted cardioverter defibrillator (ICD)	☐ Yes	☐ No	☐ Unknown
Any Cardiac stent/ pulmonary coils	☐ Yes	☐ No	☐ Unknown
Electronic implant or device	☐ Yes	☐ No	☐ Unknown
Magnetically-activated implant or device	☐ Yes	☐ No	☐ Unknown
Neurostimulation system	☐ Yes	☐ No	☐ Unknown
Any type of prosthesis (eye, penile, etc.)	☐ Yes	☐ No	☐ Unknown
External electrodes	☐ Yes	☐ No	☐ Unknown
Heart valve prosthesis	☐ Yes	☐ No	☐ Unknown
Eyelid spring or wire	☐ Yes	☐ No	☐ Unknown
Artificial or prosthetic limb	☐ Yes	☐ No	☐ Unknown
Metallic stent, filter, or coil	☐ Yes	☐ No	☐ Unknown
Shunt (spinal or intraventricular)	☐ Yes	☐ No	☐ Unknown
Vascular access port and/or catheter	☐ Yes	☐ No	☐ Unknown
Radiation seeds or implants	☐ Yes	☐ No	☐ Unknown
Swan-Ganz or thermodilution catheter	☐ Yes	☐ No	☐ Unknown
Medication patch (Nicotine, Nitroglycerine, etc.)	☐ Yes	☐ No	☐ Unknown
Any metallic fragment or foreign object	☐ Yes	☐ No	☐ Unknown
Wire mesh implant	☐ Yes	☐ No	☐ Unknown
Tissue expander (e.g., breast)	☐ Yes	☐ No	☐ Unknown
Surgical staples, clips, or metallic sutures	☐ Yes	☐ No	☐ Unknown
Joint replacement (hip, knee, etc.)	☐ Yes	☐ No	☐ Unknown
Bone/joint pin, screw, nail, wire, plate, etc.	☐ Yes	☐ No	☐ Unknown
Dentures or partial plates	☐ Yes	☐ No	☐ Unknown
Tattoo or permanent makeup	☐ Yes	☐ No	☐ Unknown
Body piercing jewelry	☐ Yes	☐ No	☐ Unknown
Hearing aids	☐ Yes	☐ No	☐ Unknown
Temperature probe (rectal, esophageal)	☐ Yes	☐ No	☐ Unknown
Temperature sensing Foley catheter	☐ Yes	☐ No	☐ Unknown
Breathing problem or motion disorder	☐ Yes	☐ No	☐ Unknown
Claustrophobia	☐ Yes	☐ No	☐ Unknown
Braces or any orthodontic implants	☐ Yes	☐ No	☐ Unknown

Spinal cord stimulator	☐ Yes	☐ No	☐ Unknown
Internal electrodes or wires	☐ Yes	☐ No	☐ Unknown
Bone growth/bone fusion stimulator	☐ Yes	☐ No	☐ Unknown
Cochlear, otologic, or other ear implants including ear tubes	☐ Yes	☐ No	☐ Unknown
Insulin or other infusion pump	☐ Yes	☐ No	☐ Unknown
Implanted drug infusion device	☐ Yes	☐ No	☐ Unknown
Tracheostomy	☐ Yes	☐ No	☐ Unknown
Other implant ____________	☐ Yes	☐ No	☐ Unknown

Please mark on the figure(s) below the location of any implant or metal inside of or on the patient's body.

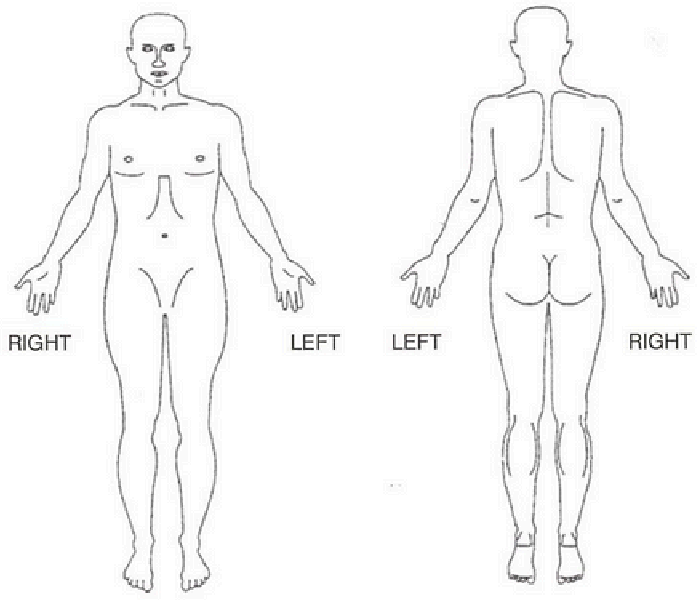

OUTPATIENT MAGNETIC RESONANCE IMAGING (MRI) PROCEDURE SCREENING

***If the answer to any of the questions on page 1 is "yes", please provide the make, model and serial number of any devices. Also provide date device was placed and surgical notes if available. If this information is unknown, write in "Notes" section.**

Device Model	Date Placed	Serial # (If known)	Notes

The above information and information on page 1 is correct to the best of my knowledge.

Signature of Person Completing Form: ______________________ Relationship to Patient: ______________

Printed Name: ______________________________ Date: ____________ Time: __________

• •

The following information is completed by MRI staff

Allergies/Adverse Reactions reviewed in EHR	☐ Yes	☐ No
Pregnant	☐ Yes	☐ No
IUD, diaphragm, or pessary	☐ Yes	☐ No
Clothes	☐ Yes	☐ No
Procedure explained	☐ Yes	☐ No
Acoustic noise - use of ear plugs or other hearing protection	☐ Yes	☐ No
Active Infection/Isolation precautions reviewed in EHR	☐ Yes	☐ No

______________________ ______________________ ________/________

MRI Staff Signature   Printed Name   Date   Time

Signature of MRI Staff who reviewed and followed-up on information including "unknown" responses, "yes" responses, and "MRI staff only" information on page 2.

Form/Information Reviewed in Radiology by:

______________________ ______________________/______________ ________/________

Signature   Printed Name   and/or   Contact Number   Date   Time

☐ MRI Technologist   ☐ Nurse   ☐ Radiologist   ☐ Other: ______________________
